# Suppression of NF-κB and NF-κB-Regulated Gene Expression by Apigenin through IκBα and IKK Pathway in TRAMP Mice

**DOI:** 10.1371/journal.pone.0138710

**Published:** 2015-09-17

**Authors:** Sanjeev Shukla, Eswar Shankar, Pingfu Fu, Gregory T. MacLennan, Sanjay Gupta

**Affiliations:** 1 Department of Urology, Case Western Reserve University, Cleveland, Ohio, United States of America; 2 The Urology Institute, University Hospitals Case Medical Center, Cleveland, Ohio, United States of America; 3 Department of Epidemiology & Biostatistics, Case Western Reserve University, Cleveland, Ohio, United States of America; 4 Department of Pathology, Case Western Reserve University, Cleveland, Ohio, United States of America; 5 Department of Nutrition, Case Western Reserve University, Cleveland, Ohio, United States of America; 6 Division of General Medical Sciences, Case Comprehensive Cancer Center, Cleveland, Ohio, United States of America; 7 Department of Urology, Louis Stokes Cleveland Veterans Affairs Medical Center, Cleveland, Ohio, United States of America; Henry Ford Health System, UNITED STATES

## Abstract

Aberrant Nuclear Factor-κappaB (NF-κB) activation due to rapid IκBα turnover and high basal IκBα kinase (IKK) activity has been frequently observed in prostate cancer. Apigenin, a naturally occurring plant flavone, exhibits anti-proliferative, anti-inflammatory and anti-carcinogenic activities by inhibiting NF-κB pathway, through a mechanism not fully understood. We found that apigenin feeding in microgram doses (bioavailable in humans) inhibited prostate tumorigenesis in TRAMP mice by interfering with NF-κB signaling. Apigenin feeding to TRAMP mice (20 and 50 μg/mouse/day, 6 days/week for 20 weeks) exhibited significant decrease in tumor volumes of the prostate and completely abolished metastasis, which correlated with inhibition of NF-κB activation and binding to the DNA. Apigenin intake blocked phosphorylation and degradation of IκBα by inhibiting IKK activation, which in turn led to suppression of NF-κB activation. The expression of NF-κB-regulated gene products involved in proliferation (cyclin D1, and COX-2), anti-apoptosis (Bcl-2 and Bcl-xL), and angiogenesis (vascular endothelial growth factor) were also downregulated after apigenin feeding. These events correlated with the induction of apoptosis in tumor cells, as evident by increased cleaved caspase-3 labeling index in the dorsolateral prostate. Our results provide convincing evidence that apigenin inhibits IKK activation and restores the expression of IκBα, preventing it’s phosphorylation in a fashion similar to that elicited by IKK and proteasomal inhibitors through suppression of NF-κB signaling pathway.

## Introduction

Prostate cancer remains the most common non-cutaneous malignancy and the second leading cause of cancer-related death among men in the United States [1, 2]. According to an estimate by the American Cancer Society, 220,800 new cases of prostate cancer will be diagnosed in the United States and 27,540 men will die of this disease in 2015 [2]. Prostate cancer-related mortality is associated with aggressive behavior to tumor cells that exhibit invasiveness, metastasis and recurrence even after definitive therapies like surgery and radiation [3, 4]. Therefore, understanding the molecular mechanism(s) and key drivers of disease progression is crucial for development of effective therapeutic approaches for treatment of this deadly form of cancer.

The Nuclear Factor-κappaB (NF-κB) signaling pathway has been implicated in increased survival and proliferation through the induction of transcription of target genes, whose product inhibit components of programmed cell death [5–7]. NF-κB consists of homodimers and heterodimers formed by several subunits: NF-κB1 (p50/p105); NF-κB2 (p52/100); RelA (p65); RelB; and c-Rel proteins [7, 8]. The NF-κB proteins are regulated by inhibitors of the IκB family, which includes IκBα, IκBβ, IκBε, IkBγ, Bcl-3, p100, and p105 [8]. In an inactive state, NF-κB is present in the cytoplasm as a heterodimer composed of p65 and p50, bound to IκB subunits. Many different stimuli cause nuclear localization and transcriptional activation of NF-κB by activation of the IKK complex resulting in phosphorylation of the IκBα subunit at serine residues 32 and 36, triggering its ubiquitination and proteasomal degradation [6–8]. Phosphorylation of p65/RelA facilitates its binding to a specific sequence in DNA, which triggers the transcriptional activation of NF-κB-regulated genes including, anti-apoptotic genes (cIAP, survivin, Bcl2, and BCl-xL), cell cycle-regulatory genes (cyclin D1), genes encoding adhesion molecules, chemokines, inflammatory cytokines, pro-angiogenic gene, vascular endothelial growth factor (VEGF), and genes involved in tumor metastasis such as cyclooxygenase-2 (COX-2), inducible nitric oxide synthase (iNOS), and matrix metalloproteinase-9 (MMP-9) [8, 9].

Aberrant NF-κB activation has been implicated in the pathogenesis of several humanmalignancies including hematological malignancies and cancers of the breast, colon, skin, lung, esophagus, uterine cervix, pancreas and prostate [10–16]. Studies have reported that NF-κB is constitutively activated in human prostate cancer tissue, androgen-refractory human prostate carcinoma cells, prostate tumor xenografts and in the prostate of the transgenic adenocarcinoma of the mouse prostate (TRAMP) model, which emulates progressive forms of human prostate cancer [17–20]. Strong nuclear NF-κB staining has been observed in prostate cancer lymph node metastases and in subsets of castrate-resistant prostate cancer patients [21]. Increase NF-κB expression correlate with disease progression and its nuclear localization is predictive of biochemical recurrence and poor survival [22, 23]. Thus, inhibition of NF-κB activation by pharmacological agents may be potentially useful in the prevention and/or treatment of prostate cancer. In recent years, suppression of NF-κB activation by plant-derived phytochemicals has been the focus of much cancer research [24, 25].

Apigenin (4′, 5, 7-trihydroxyflavone) is a flavone present in common fruits, vegetables, herbs and spices. It is abundantly present in plants such as celery, parsley and dried chamomile flowers [26]. Apigenin has been shown to suppress tumorigenesis, inhibit tumor promotion, and suppress angiogenesis [26–28]. Several of these effects of apigenin are mediated through suppression of the expression of COX-2, iNOS, MMP-9 and cyclinD1 [29, 30], all of which are genes regulated by NF-κB. In addition, apigenin has been shown to induce apoptosis in a variety of cancer cells including breast, melanoma, thyroid, skin, hepatoma, prostate carcinoma and lymphoma, acute myelogeous leukemia [31–37], through inhibition of DNA replication, caspase activation, perturbance in cell cycle regulatory molecules, inhibition of protein kinases, generation of reactive oxygen species, mitochondrial damage and interference in Ku70-Bax interaction [38–41]. Another mechanism by which apigenin induces apoptosis involves downregulation of the inhibitor of apoptosis (IAP) family of genes, another gene known to be regulated by NF-κB. Its anti-tumorigenic, anti-angiogenic and pro-apoptotic effects combined with its ability to suppress the expression of COX-2, iNOS, MMP-9, VEGF, cyclinD1 and the IAP family proteins suggest that apigenin mediates its effects through suppression of NF-κB [30, 41].

In the present study, we investigated in detail the effect of oral intake of apigenin at doses equivalent to human consumption of a healthy diet of flavonoids (6–64mg/day of flavones and flavonols) in transgenic adenocarcinoma of the mouse prostate (TRAMP) mice [42]. We report that NF-κB inactivation by apigenin occurs at various levels. Apigenin in a sequential manner causes inactivation and downregulation of IKKα preventing IκBα degradation, reduced expression of p65/p50 and cessation of DNA binding of NF-κB/p65. Inhibition of these events caused lower expression of NF-κB-regulated genes, resulting in suppression of prostate carcinogenesis in TRAMP mice.

## Materials and Methods

### Animals

Male and female heterozygous C57BL/TGN TRAMP mice, Line PB Tag 8247NG, were purchased as breeding pairs from The Jackson Laboratory (Ann Arbor, MI). The animals were bred and maintained at the Association for Assessment and Accreditation of Laboratory Animals-accredited Animal Resource Facility of Case Western Reserve University. Housing and care of the animals were in accordance with the guidelines established by the University’s Animal Research Committee and with the National Institutes of Health Guidelines for the Care and Use of Laboratory Animals. The protocol was approved by the Institutional Animal Care and Use Committee (IACUC) on the Ethics of Animal Experiments of the Case Western Reserve University (Assurance Number: A3145-01). Transgenic males for the studies were routinely obtained as (TRAMP x C57BL/6) F1 or as (TRAMP x C57BL/6) F2 offspring. Identity of transgenic mice was established by PCR-based DNA screening as described previously [38].

### Animal experimentation

Approximately 8-week-old male TRAMP mice were used in the studies. The animals received autoclaved Teklad 8760 high-protein diet and tap water ad libitum throughout the study. Apigenin (10 mg) was suspended in 1 ml vehicle material (0.5% methyl cellulose and 0.025% Tween 20) by sonication for 30 sec at 4°C and further diluted for appropriate concentration. Apigenin at 20 and 50 μg/mouse/day (wt/vol) was administered by gavage in 0.2 ml of a vehicle consisting of 0.5% methyl cellulose and 0.025% Tween 20 to TRAMP mice beginning at 8 weeks of age and was continued until the animals were 28 weeks old, at which time the experiment was terminated. These doses are comparable with the daily consumption of flavonoid in humans as reported in previously published studies [38, 43].

### Tissue preparation and histology

The genitourinary (GU) apparatus was isolated followed by excision of the dorsolateral and ventral prostate and weighed separately. A small portion of the dorsolateral and ventral prostates was fixed overnight in 10% zinc-buffered formalin and then transferred to 70% ethanol. Sections (4 μm) were cut from paraffin-embedded tissue and mounted on slides. The sections were stained with hematoxylin and eosin as described previously [38, 43] and were evaluated for the presence or absence of the following lesions: well-differentiated adenocarcinoma, moderately differentiated adenocarcinoma and poorly differentiated adenocarcinoma. The histological characteristics of these lesions have been well established and described in previous publications [38, 44].

### Metastasis examination

Microscopic examinations of lymph nodes, liver and lungs were performed to evaluate for the presence of metastases. The India ink method was used to examine the lungs for metastasis as described previously [38].

### Western blot analysis

Dorsolateral prostate tissue from treated and control groups were subjected to preparation of total tissue lysate and isolation of cytosolic and nuclear fractions as described previously [38]. For Western blotting, 25 μg of protein was resolved over 4–20% Tris-glycine polyacrylamide gel and then transferred onto the nitrocellulose membrane. The blots were blocked using 5% non-fat dry milk and probed using appropriate primary antibodies overnight at 4°C. The antibodies used were anti-IKKα (sc-7218), anti-IKKβ (sc-34673), anti-NF-κB/p65 (sc-8008), anti-NF-κB/p50 (sc-8414), anti-IκBα (sc-1643) anti-p-IκBα (sc-8404), anti-Bax (sc-493), anti-Bcl2 (sc-7382), anti-COX-2 (sc-795), anti-cyclin D1 (sc-533), anti-PCNA (sc-56), and anti-VEGF (sc-152) procured from Santa Cruz Biotechnology, Santa Cruz, CA. Antibodies including anti-cleaved caspase-3 (#9661), anti-Bcl-xL (#2762), anti-p-IKKα/β (#2078) were purchased from Cell Signaling Technology, Danvers, MA. Anti-β-actin (#A1978) was obtained from Sigma-Aldrich, St. Louis, MO. The membrane was then incubated with appropriate secondary mouse (sc-2005) and rabbit (sc-2004) antibody horseradish peroxidase conjugate (Santa Cruz Biotechnology) followed by detection using chemiluminescence ECL kit (GE Healthcare Biosciences). For equal loading of proteins, the membrane was probed with appropriate loading controls.

### Electrophoretic mobility shift assay (EMSA)

EMSA for NF-κB/p65 was performed using the Lightshift Chemiluminescent EMSA kit (Pierce, Rockford, IL) following the manufacturer’s protocol as previously described [20]. Briefly, DNA was biotin labeled using the biotin 3′-end-labeling kit (Pierce) in a 50-μl reaction buffer and 5 pmol of double-stranded NF-κB oligonucleotide (5′-AGTTGAGGGGACTTTCCCAGGC-3′ and 3′-TCAACTCCCCTGAAAGGGTCCG-5′) incubated in a microfuge tube with 10 μl of 5× terminal deoxynucleotidyltransferase buffer, 5 μl of 5 μm biotin-N4-CTP, 10 units of diluted terminal deoxynucleotidyltransferase, and 25 μl of ultrapure water at 37°C for 30 min. The reaction was stopped with 2.5 μl of 0.2 m EDTA. To extract labeled DNA, 50 μl of chloroform:isoamyl alcohol (24:1) were added to each tube and centrifuged at 13,000 x g. The top aqueous phase containing the labeled DNA was further used for binding reactions. Each binding reaction contained 1x binding buffer [100 mm Tris, 500 mm KCl, and 10 mm DTT (pH 7.5)], 2.5% glycerol, 5 mm MgCl2, 50 ng/μl poly(deoxyinosinic-deoxycytidylic acid), 0.05% NP40, 2.5 μg of nuclear extract, and 20–50 femtomoles of biotin-end-labeled target DNA. The contents were incubated at room temperature for 20 min. To this reaction mixture, 5 μl of 5x loading buffer were added, subjected to gel electrophoresis on a native polyacrylamide gel, and transferred to a nylon membrane. After transfer was completed, DNA was cross-linked to the membrane at 120 mJ/cm2 using a UV cross-linker equipped with a 254-nm bulb. The biotin-end-labeled DNA was detected using streptavidin-horseradish peroxidase conjugate and a chemiluminescent substrate. The membrane was exposed to X-ray film (XAR-5; Amersham Life Science Inc.) and developed using a Kodak film processor.

### Immunoprecipitation

Total cell lysate from the prostates of treated and untreated mice (200 μg) were immunoprecipitated with 2 μg appropriate primary IKKα antibody and were incubated at 4°C for 3h. Protein A/G beads (20 μl) were added and incubated overnight at 4°C. Immunoprecipitated proteins were washed four times with lysis buffer, electrophoresed by sodium dodecyl sulfate–polyacrylamide gel electrophoresis and analyzed by Western blotting.

### IKKα kinase activity assay

For this assay, 200 μg of total protein fraction from the prostates of treated and untreated mice was precleared with A-G beads and immunoprecipitated with agarose-conjugated IKKα antibody (Cell Signaling Technology), incubated with glutathione S-transferase-IκB substrate and subjected to SDS-PAGE as previously described [20].

### IκBα degradation

To determine the effect of apigenin on IκBα degradation, fresh tissue extract from the prostate of TRAMP mice was prepared and treated with 20 μM apigenin and 10 μM MG132 incubated at 37°C for 8 h. The extracts were then resolved on 10% SDS-polyacrylamide gels. After, electrophoresis, the proteins were electrotransferred to nitrocellulose membrane, probed with antibodies against anti-IκBα and anti-p-IκBα, and detected by chemiluminescence (ECL, Amersham) as previously described [30].

### Immunohistochemistry

Immunohistochemistry (IHC) for proliferation cell nuclear antigen (PCNA), p-IKKα/β, p-IκBα, NF-κB/p65, and cleaved caspase 3 was performed on formalin-fixed, paraffin-embedded prostate tissue sections using a standard protocol as described previously using 3,3′-diaminobenzidine and counterstaining with Mayer’s hematoxylin [20, 38]. Sections were examined with an inverted Olympus BX51 microscope and images were acquired with Olympus MicroSuite™ Five Software (Soft Imaging System, Lakewood, CO).

### Statistical analysis

Data were summarized as mean ± SD and visualized using bar diagram. The difference of the data including GU weight, ventral lobe prostate, dorsolateral lobe prostate among three treatment groups (control, 20 and 50 μg apigenin) was examined using analysis of variance followed by Turkey multiple comparison procedure. All tests are two tailed and *P* value <0.05 were considered to be statistically significant.

## Results

### Apigenin intake inhibits prostate carcinogenesis in TRAMP mice

**Fig 1:**
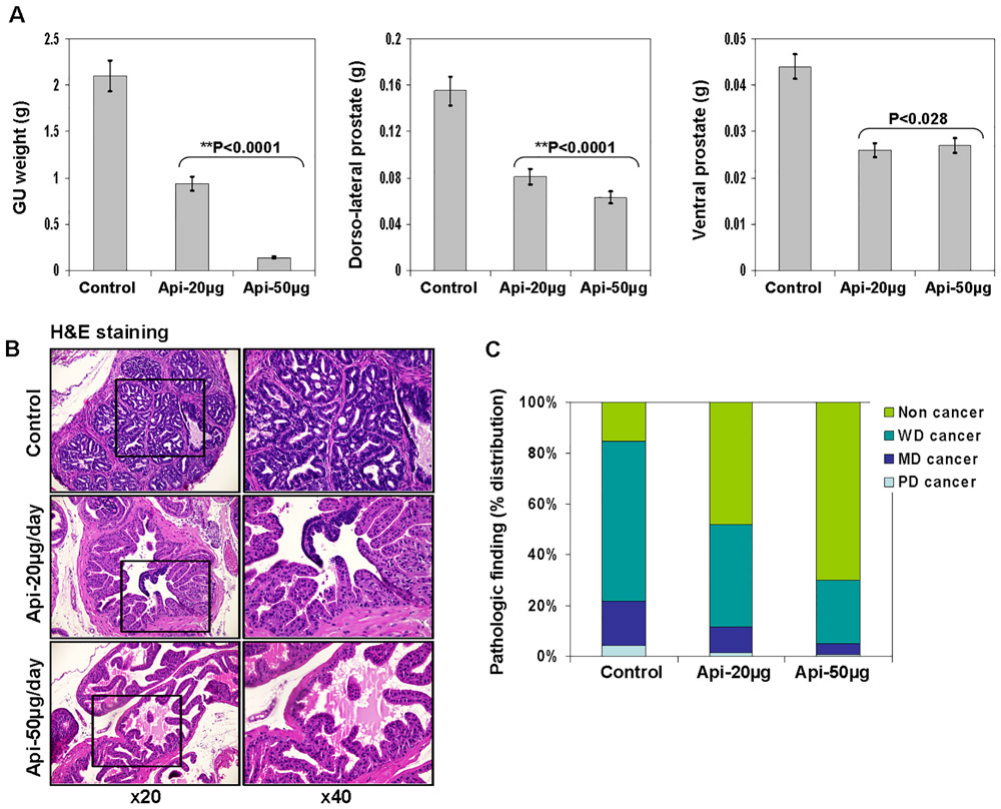
Effect of apigenin intake on prostate cancer progression in TRAMP mice. Apigenin was fed at 20 and 50 μg/mouse/day (wt/vol) by gavage in 0.2 ml of a vehicle consisting of 0.5% methyl cellulose and 0.025% Tween 20 to TRAMP mice beginning at 8 weeks of age for 20 weeks till experiment was terminated. (**A**) Weight of GU apparatus, ventral and dorsolateral prostate lobes of control and apigenin-treated TRAMP mice at 28 weeks of age. Values represent Mean + SE, **P<0.05, compared to control. (**B**) Pathologic finding evaluated in hematoxylin and eosin-stained slides. TRAMP mice (control) exhibited adenocarcinoma with extensive epithelial stratification, crowded cribriform structures accompanied with marked thickening, remodeling and hypercellularity of the fibromuscular stroma. Apigenin intake to TRAMP mice resulted in a marked reduction in epithelial stratification and cribriform structures. Magnification, ×20 and ×40. **(C)** Distribution of pathologic findings after apigenin intake in the dorsolateral lobes of TRAMP mice. Prostatic lobe was scored for percentage of each pathologic finding present in hematoxylin and eosin-stained slides in the dorsolateral lobe in TRAMP mice at 28 weeks of age. Pathologic findings: WD, well-differentiated cancer; MD, moderately differentiated cancer; PD, poorly differentiated cancer. Details are described in ‘materials and methods’ section.

The autochthonous TRAMP mice exhibit both histological and morphological features that mimic human prostate carcinogenesis [20, 44]. TRAMP mice exhibit low-grade prostatic intraepithelial neoplasia at 6 weeks that progresses to high-grade PIN by 12 weeks. Focal adenocarcinoma develops between 12 and 18 weeks and progresses to poorly differentiated carcinoma by 28 weeks. After 28 weeks, these mice develop occasional metastasis to lungs, lymph nodes, liver and bone. TRAMP mice have been widely used for the investigation of molecular targets and therapeutic agents. In our experiments, we used TRAMP/C57BL6 mice. Apigenin treatment at the doses of 20 and 50 μg/day for 6 days a week was initiated when the mice were of 8 weeks of age. Control group of mice received vehicle only. No apparent toxicity or loss of weight was observed with apigenin administration during the entire period of the experiment. All the mice were examined at 28 weeks of age for the growth of prostate cancer [38, 43]. As shown in Fig 1A, apigenin intake for 20 weeks elicited a significant decrease in GU weight (*P*<0.0001) and in the dorsolateral (*P*<0.0001) and ventral prostate (*P*<0.028), compared with the control group.

### Apigenin intake inhibits progression and aggressiveness of adenocarcinoma in TRAMP mice

Histopathological analysis of the excised prostate tumor tissues of both control and apigenin treatment at 20 and 50 μg doses for 20 weeks is shown in Fig 1B and 1C. Composite analysis of the prostates of control group mice at 28 weeks of age exhibited predominantly well-differentiated adenocarcinoma (63.9%), along with moderately-differentiated cancer (17.95%) and, infrequently, poorly-differentiated adenocarcinoma (4.1%) in the prostate. Approximately 15.18% of the prostate tissue did not exhibit detectable cancer in control mice. Four of six mice (66.6%) in the control group exhibited metastasis to lymph nodes, whereas one mouse showed liver (16.6%) and lung metastases (16.6%), respectively. At 28 weeks of age, the histological findings in the prostates of TRAMP mice given 20 μg of apigenin per day were notably different from findings in the control group, showing a decrease in the percentage of well-differentiated carcinoma (40.8%), moderately-differentiated cancer (10.04%) and poorly-differentiated cancer (1.52%). Approximately 48.5% of the prostate tissue of TRAMP mice given 20 μg of apigenin per day appeared to be non-cancerous. The prostates of TRAMP mice receiving a higher dose of 50 μg apigenin exhibited 25.1% of well-differentiated cancer, 4.52% of moderately-differentiated cancer and 0.478% of poorly-differentiated cancer, and ~70.2% of the prostate tissue appeared to be non-cancerous (Fig 1B and 1C). No metastases were recorded in any of the mice treated with apigenin. These data suggest the inhibitory potential of apigenin against prostate cancer progression.

### Apigenin intake suppresses NF-κB activation in the prostate of TRAMP mice

**Fig 2:**
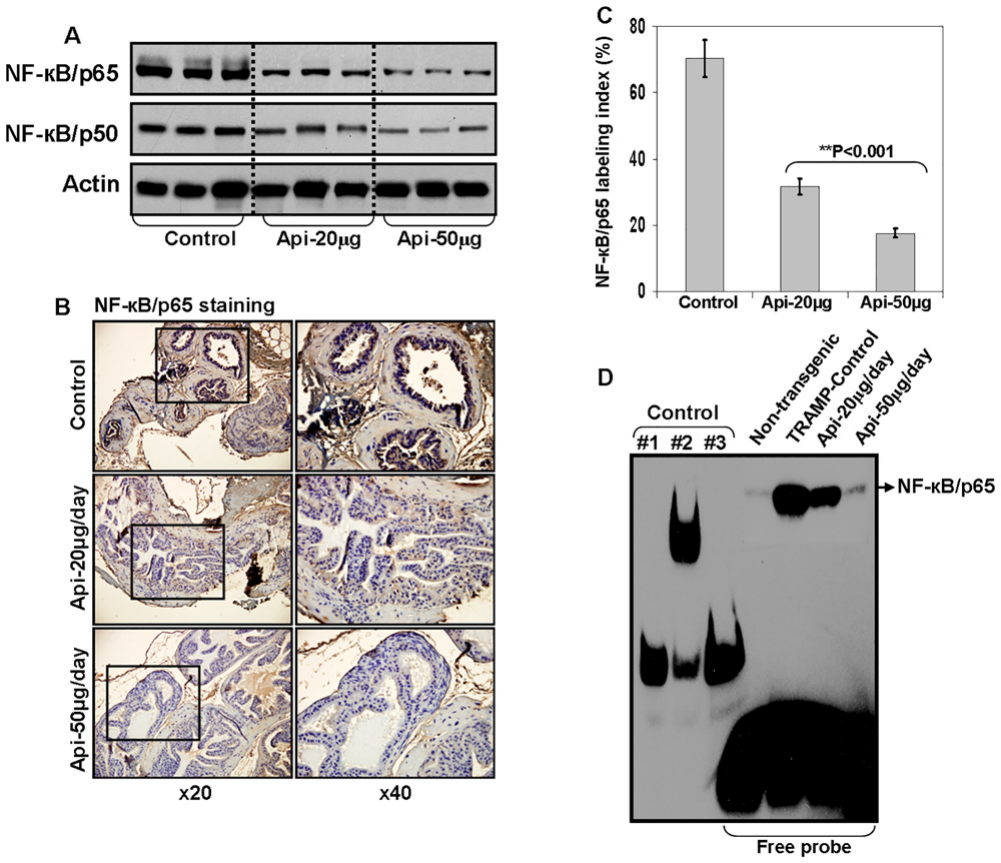
Effect of apigenin intake on NF-κB activation in the dorsolateral prostate of TRAMP mice. **(A)** Protein expression of NF-κB/p65 and NF-κB/p50 as determined by Western blot analysis. A significant decrease in NF-κB/p65 and NF-κB/p50 is observed after apigenin intake. Actin as loading control. **(B)** IHC of NF-κB/p65 in the dorsolateral prostate from control and apigenin-treated TRAMP mice. A marked decrease in the expression of NF-κB/p65 is observed after apigenin intake. **(C)** NF-κB/p65 labeling index was calculated as number of NF-κB/p65 positive cells x 100 / total number of cells counted under x40 magnification in four randomly selected areas in each sample. Values represent Mean + SE, **P<0.05, compared to control. **(D)** DNA binding assay by EMSA. Nuclear extracts were prepared, and electrophoretic mobility shift assay was performed to assess NF-κB DNA binding activity. A marked decrease in the DNA binding of NF-κB/p65 is observed after apigenin intake. Controls were as follows: #1, biotin-Epstein-Barr virus Nuclear Antigen (EBNA) control DNA; #2, biotin-EBNA control DNA + EBNA extract; #3, biotin-EBNA control DNA + EBNA extract + 20-fold molar excess of unlabeled EBNA DNA. Details are described in ‘materials and methods’ section.

Next, we evaluated NF-κB expression in apigenin-mediated growth suppressive effects on the dorso-lateral prostate of TRAMP mice. As shown in Fig 2A, apigenin feeding to TRAMP mice inhibited NF-κB activation in dose-dependent fashion. The levels of NF-κB/p65 was markedly reduced at 50μg/day dose of apigenin. The inhibition in the level of NF-κB/p50 was of a lower magnitude after apigenin intake. These results were further confirmed by IHC analysis of prostate tissue stained for NF-κB/p65 where a significant decrease in the NF-κB/p65 labeling index was noted after apigenin feeding (Fig 2B and 2C).

To determine whether apigenin intake interferes with NF-κB binding to the DNA, nuclear lysate were prepared from the control and treated groups and DNA binding activity was performed using EMSA. As shown in Fig 2D, apigenin supplementation at 20- and 50- μg doses resulted in significant decrease in NF-κB/p65 binding to the DNA, compared with the control group.

### Apigenin intake inhibits phosphorylated IKKα expression in the prostate of TRAMP mice

**Fig 3:**
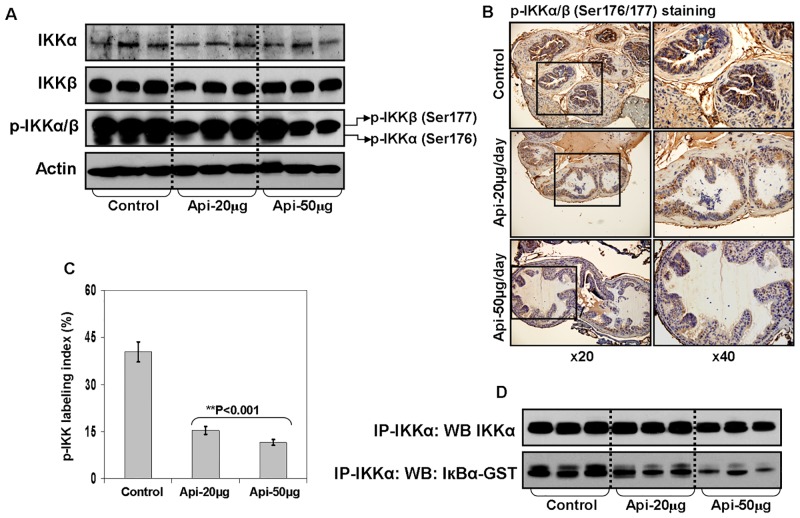
Effect of apigenin intake on IKK activation in the dorsolateral prostate of TRAMP mice. **(A)** Protein expression of IKKα, IKKβ and its phosphorylation as determined by Western blot analysis. A significant decrease in p-IKKα levels is observed after apigenin treatment. Actin as loading control. **(B)** IHC of p-IKKα/β in the dorsolateral prostate from control and apigenin-fed TRAMP mice. A marked decrease in the expression of p-IKKα/β is observed after apigenin intake. **(C)** p-IKKα/β labeling index was calculated as number of p-IKK positive cells x 100 / total number of cells counted under x40 magnification in four randomly selected areas in each sample. Values represent Mean + SE, **P<0.05, compared to control. **(D)** IKKα kinase activity. Total protein from control and apigenin-fed group were immunoprecipitated with agarose-conjugated IKKα antibody overnight, and kinase assay by determining phosphorylated expression of IκBα were performed using IκBα-GST as substrate. A marked decrease in IKK activity is observed after apigenin intake. Details are described in ‘materials and methods’ section.

Strong IKKα/β phosphorylation at Serine176/180 was observed in human prostate cancer specimens [45]. We have previously shown increase in kinase activity of IKKα in the TRAMP mouse prostate in age-dependent fashion which leads to increased phosphorylation and faster turnover of IκBα in the cytosol, correlating with cancer progression [20]. To determine whether apigenin targets the expression of IKKα/β and its phosphorylation during prostate cancer progression in TRAMP mice, we determined the expression of IKKα, IKKβ and its phosphorylation. IKKα kinase activity by western blotting and IHC in excised prostate tissue from control and apigenin-treated TRAMP mice was assessed. Apigenin intake of TRAMP mice resulted in decrease p-IKKα/β (Serine176/177) expression in the prostate tissue, compared to the control group. A marked decrease in p-IKKα (Serine176) was noted after 50 μg apigenin feeding, compared to p-IKKβ at Serine177 (Fig 3A). These results were further confirmed by IHC analysis for tissue staining for p-IKKα/β (Fig 3B and 3C). No significant changes were noted on the protein levels of total IKKα and IKKβ.

### Apigenin intake decreases IKKα kinase activity in the prostate of TRAMP mice

In an attempt to understand whether the inhibitory effect of apigenin on IKK activity is a direct response or is mediated via an upstream event, we next performed an *in vitro* IKKα kinase activity assay using fresh tissue lysates from control and apigenin-fed groups. For this assay, equal amount of lysate from various groups were immunoprecipitated with agarose conjugated IKKα antibody and the immuno-complex was subjected to kinase assay after incubation with IκBα-GST as a substrate. Interestingly, a decrease in IKKα kinase activity was observed in apigenin-fed group in dose-dependent fashion, compared to the control group (Fig 3D). This observation suggests that the inhibitory effect of apigenin on IKKα kinase activity is rather a direct response.

### Apigenin intake inhibits phosphorylation and degradation of IκBα in the prostate of TRAMP mice

**Fig 4:**
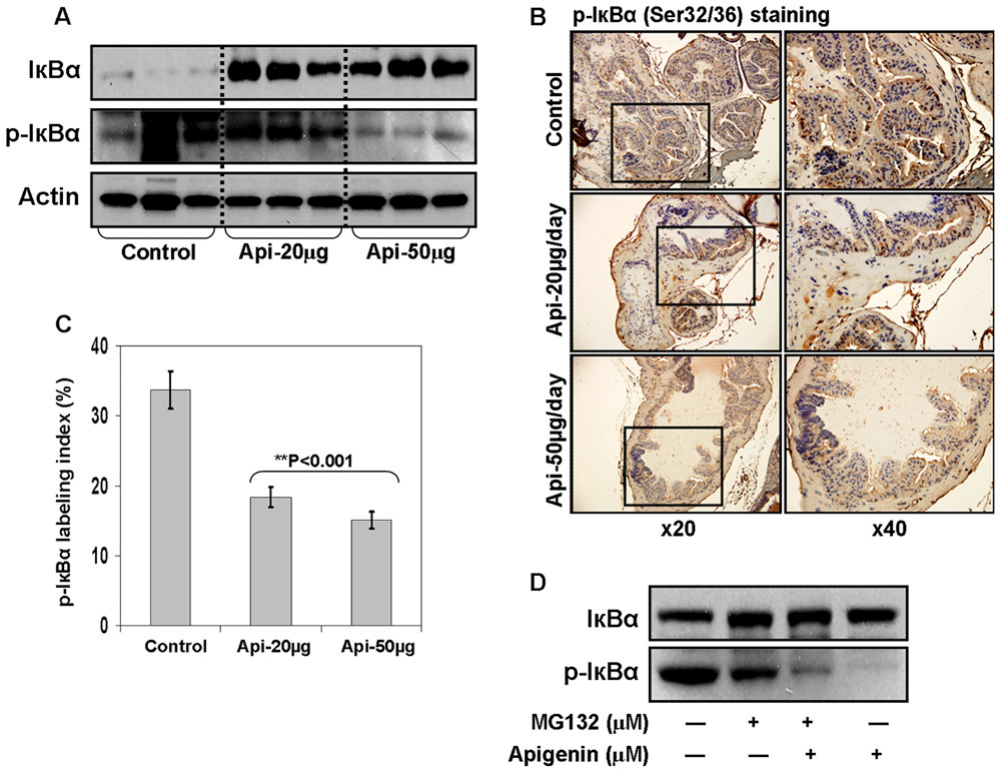
Effect of apigenin intake on IκBα levels in the dorsolateral prostate of TRAMP mice. **(A)** Protein expression of IκBα and its phosphorylation as determined by Western blot analysis. A significant increase in total IκBα and a marked decrease in p-IκBα levels is observed after apigenin intake. Actin as loading control. **(B)** IHC of p-IκBα in the dorsolateral prostate from control and apigenin-treated TRAMP mice. A marked decrease in the expression of p-IκBα is observed after apigenin treatment. **(C)** p-IκBα labeling index was calculated as number of p-IκBα positive cells x 100 / total number of cells counted under x40 magnification in four randomly selected areas in each sample. Values represent Mean + SE, **P<0.05, compared to control. **(D)** IκBα degradation by blocking IκBα phosphorylation. Tissue extract of control group was treated with either DMSO or 20 μm apigenin, or 10 μM MG132 or both for 8 h at 37°C followed by Western blot analysis of IκBα and p-IκBα. Apigenin blocked IκBα degradation and inhibited its phosphorylation. Details are described in ‘materials and methods’ section.

The translocation of NF-κB to the nucleus is preceded by the phosphorylation, ubiquitination and proteolytic degradation of IκBα. To determine whether apigenin blocks phosphorylation of IκBα, we examined the total and phosphorylated levels of IκBα in the dorso-lateral prostate of TRAMP mice treated with vehicle and 20- and 50- μg apigenin. As shown in Fig 4A, apigenin intake to TRAMP mice resulted in marked increase in the protein expression of IκBα at both doses, whereas a significant decrease in p-IκBα was noted after 50 μg apigenin intake. These results were further confirmed by IHC analysis of prostate tissue stained for p-IκBα where a significant decrease in p-IκBα labeling index was noted after apigenin intake, compared to the control group (Fig 4B and 4C).

To further confirm whether the observed inhibitory effect of apigenin on NF-κB is from blocking IκBα phosphorylation, we used MG132, a proteasomal inhibitor belonging to the class of peptide aldehydes that potently but reversibly inhibits chymotrypsin-like activity of the proteasome. As shown in Fig 4D, increase in the level of total IκBα was observed on treatment with either MG132 and apigenin or combination of the two. With regard to p-IκBα levels in the experiment, no apparent change in the protein expression of p-IκBα was noted after MG132 exposure, however apigenin treatment resulted in significant decrease in p-IκBα, which is partially rescued by simultaneous addition with MG132. These results are indicative of the inhibitory effect of apigenin on the phosphorylation and degradation of IκBα.

### Apigenin intake inhibits NF-κB downstream target proteins in the prostate of TRAMP mice

**Fig 5:**
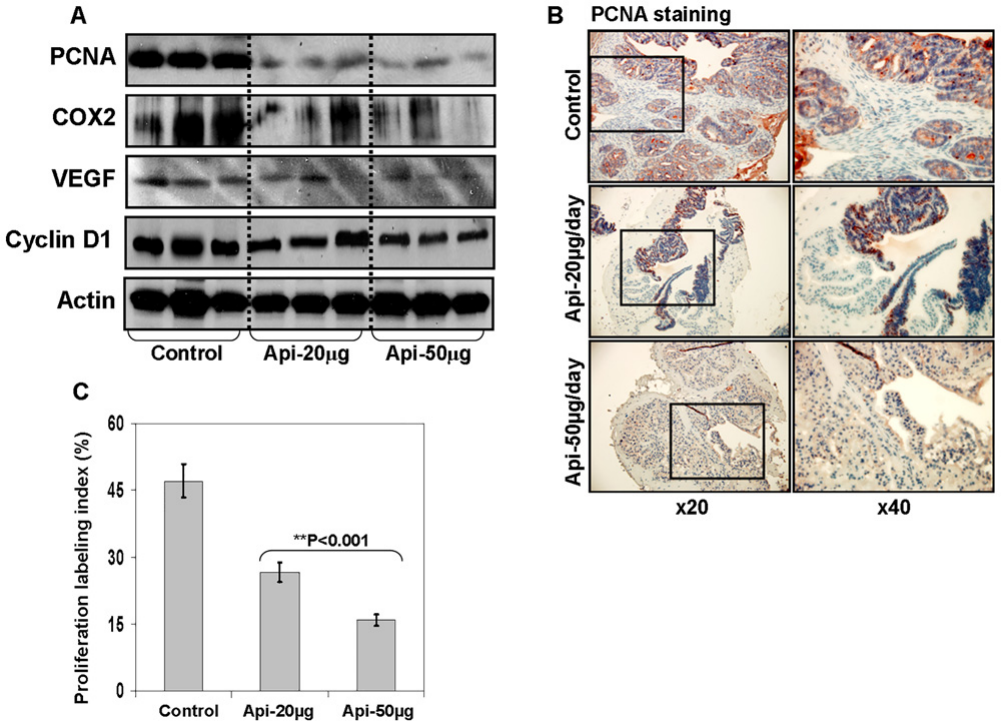
Effect of apigenin intake on proliferation and NF-κB-regulated genes in the dorsolateral prostate of TRAMP mice. **(A)** Protein expression of PCNA, COX-2, VEGF, cyclin D1 as determined by Western blot analysis. A significant decrease in PCNA, COX-2, VEGF, cyclin D1 level is observed after apigenin intake. Actin as loading control. **(B)** IHC of PCNA in the dorsolateral prostate from control and apigenin-fed TRAMP mice. A marked decrease in proliferation as assessed by PCNA expression is observed after apigenin intake. **(C)** Proliferation labeling index was calculated as number of PCNA positive cells x 100 / total number of cells counted under x40 magnification in four randomly selected areas in each sample. Values represent Mean + SE, **P<0.05, compared to control. Details are described in ‘materials and methods’ section.

Next, to gain insight into the NF-κB-mediated mechanism(s) underlying *in vivo* anticancer efficacy of apigenin, we determined the expression of various NF-κB responsive genes including COX-2, VEGF, cyclin D1, Bcl2 and Bcl-xL along with assessment of proliferation and apoptotic response. We assessed proliferation through a ubiquitous molecular maker of proliferation i.e. proliferating cell nuclear antigen (PCNA) which interacts with the cyclin-cdk complex during cell cycle progression and is absent in the resting G0-phase cells. As shown in Fig 5A, apigenin feeding to TRAMP mice resulted in marked decrease in the protein expression of PCNA, COX-2, VEGF, and cyclin D1, compared to the control group. IHC analysis indicated that apigenin treatment inhibited PCNA expression in the prostate of TRAMP mice thereby reducing the proliferative index (Fig 5B and 5C).

**Fig 6:**
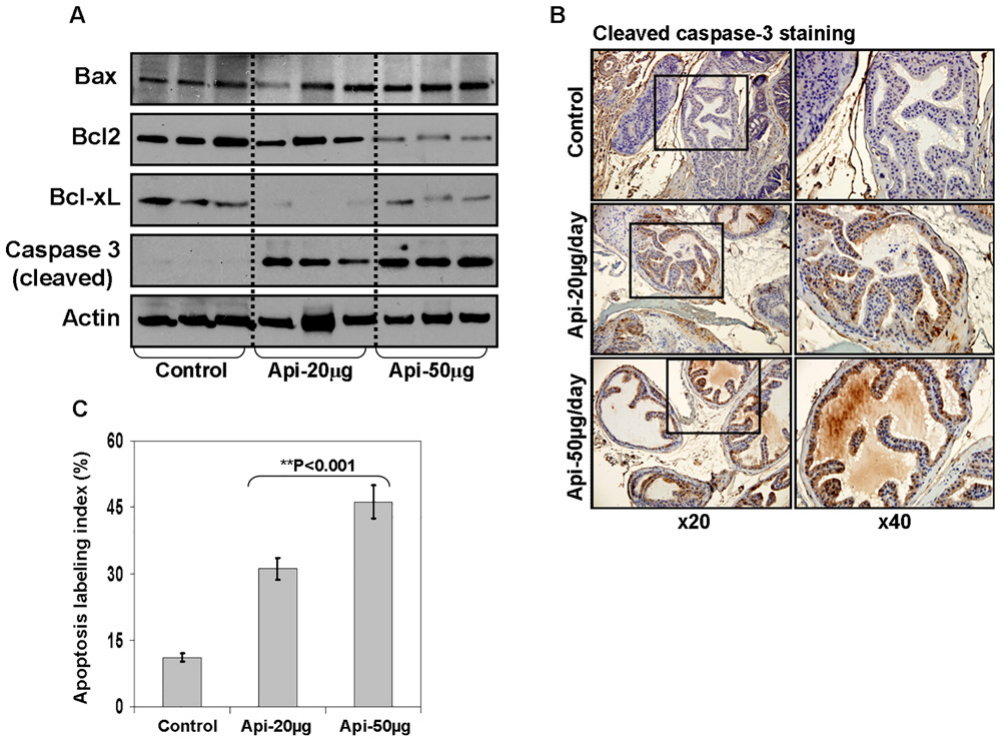
Effect of apigenin intake on NF-κB-regulated anti-apoptotic and pro-apoptotic genes in the dorsolateral prostate of TRAMP mice. **(A)** Protein expression of Bax, Bcl2, Bcl-xL, and cleaved caspase 3 as determined by Western blot analysis. A significant decrease in Bcl2, and Bcl-xL levels; whereas an increase in Bax and cleaved caspase 3 expression is observed after apigenin intake. Actin as loading control. **(B)** IHC of cleaved caspase 3 in the dorsolateral prostate from control and apigenin-fed TRAMP mice. A marked induction of apoptosis is observed after apigenin treatment. **(C)** Apoptosis index was calculated as number of cleaved caspase 3 positive cells x 100 / total number of cells counted under x40 magnification in four randomly selected areas in each sample. Values represent Mean + SE, **P<0.05, compared to control. Details are described in ‘materials and methods’ section.

We also determine the effect of apigenin feeding on Bcl2 family members and cleaved caspase 3, as a marker of apoptosis. As shown in Fig 6A, apigenin feeding to TRAMP mice resulted in increase in the levels of Bax, whereas a simultaneous decrease in the protein expression of Bcl2 and Bcl-xL was observed as a result of apigenin intake. Bcl2 and Bcl-xL are NF-κB regulated genes. Our results demonstrate that apigenin alters the Bax/Bcl2 ratio to shift in favor of apoptosis in tumor cells. These results were further evident from increase in the expression of cleaved caspase 3 by IHC, a marker of apoptotic cell death (Fig 6B and 6C).

## Discussion

Apigenin has been shown to possess anti-inflammatory, immune-modulatory, anti-proliferative and antioxidant properties in cell culture and in various in vivo models [26–28]. In the present study we demonstrate tumor growth suppression and complete inhibition of metastasis by apigenin intake are associated with inhibition of DNA binding activity of NF-κB/p65, IκBα degradation, IκBα phosphorylation, IKK activation and NF-κB-dependent gene expression in the dorsolateral prostate of TRAMP mice. Apigenin-mediated inhibition of NF-κB activation correlated with suppression of NF-κB dependent cyclin D1, COX-2, VEGF, Bcl2 and Bcl-xL expression. These results are in line with reports suggesting the pathophysiologic role of NF-κB in prostate cancer progression. Our previous studies have shown that apigenin suppresses NF-κB activation in prostate cancer cells and sensitize them to TNFα-mediated apoptosis and we further validated our hypothesis in *in vivo* model [30]. We and others have previously shown that NF-κB is constitutively activated in human prostate adenocarcinoma and its nuclear localization correlates with disease progression [17–19]. Compared with benign prostate tissue, higher NF-κB levels are detected in low- and high- grade prostate cancer specimens and are associated with the expression of NF-κB–regulated gene products including Bcl2, cyclinD1, MMP-9, and VEGF. Constitutive NF-κB activation represents an independent risk factor for prostate cancer recurrence and poor survival [22, 23]. Higher NF-κB expression is also observed in human prostate cancer cell lines and in xenograft models, where greater constitutive NF-κB levels is consistently observed in androgen-refractory prostate cancer cells, compared to androgen-responsive counterparts [19]. Above all, we have shown a progressive increase in NF-κB activity and its dependent gene products in TRAMP mice, suggesting that these molecules and their interrelated pathways may be worthwhile therapeutic targets [20]. Pharmacologic and genetic inhibition of NF-κB results in decrease invasion, angiogenesis, clonogenicity, tumorigenicity and metastasis in various pre-clinical models of prostate cancer [46–48]. In agreement with these results, prostates from apigenin-supplemented TRAMP mice showed marked suppression of tumorigenecity and metastatic activity, which correlated with a decrease in NF-κB/p65 activation and its binding to the DNA in the dorsolateral prostate.

Altered expression of IκBα in prostate cancer specimens has been linked to constitutive NF-κB activation through phosphorylation of IκBα at Ser32/36, resulting in the release and nuclear translocation of NF-κB [17–20]. An increase in IκBα phosphorylation has been observed in cancer specimens, compared to benign tissue, which is mediated by upstream kinase IKKs, including IKKα and IKKβ [17, 45]. Pharmacological inhibitors of IKK and proteasomal inhibitors have received increasing attention in recent years, because of their demonstrated ability to inhibit IKK and to induce apoptosis in cancer cells, findings that are well documented in both pre-clinical and clinical studies [47]. Our studies indicate that apigenin inhibits IKK activation by suppressing its kinase activity and inhibits IκBα phosphorylation and degradation by abrogation of DNA binding of NF-κB and gene transcription. How apigenin suppresses IKK activation remains unclear. It is possible that apigenin physically binds with IKKα/β in the active site to suppress its activity, without affecting the protein levels. This remains an area of further investigation.

Reports suggest that PI3K/Akt and NIK primarily activate IKKα, whereas MEKK1 and atypical protein kinase C activate IKKβ [5–8]. In our studies, apigenin intake by TRAMP mice inhibited IKK activity in the dorsolateral prostate without directly interfering with the IKK protein. Thus, it is possible that apigenin-mediated IKK blockade might be due to inhibition of one or many of the upstream kinases responsible for IKK activation. In this regard, we have recently demonstrated that apigenin targets the PI3K/Akt pathway in suppression of prostate cancer progression in autochthonous transgenic mouse model of prostate cancer [38]. Further detailed studies are warranted to evaluate the effect of apigenin on various kinases and to elucidate the mechanisms of action.

Accumulated data indicate that NF-κB regulates a wide variety of genes whose products are involved in proliferation, inflammation, carcinogenesis, and evasion of apoptosis [5–8]. Constitutive activation of NF-κB may up-regulate the expression of inflammatory cytokines, chemokines, cell adhesion molecules, and inflammatory gene products such as COX-2 and iNOS [8, 9]. Anti-apoptotic genes that are regulated by NF-κB include genes encoding Bcl2-like proteins viz. Bcl2 and Bcl-xL and inhibitor of apoptosis proteins including cIAP1, cIAP2, XIAP and survivin [8, 9]. Moreover, pro-metastatic genes such as interleukin 6, urokinase plasminogen activator, MMP-9, interleukin 8, and VEGF are induced by NF-κB [8, 9]. It has been shown recently that NF-κB is an important regulator of cell proliferation by its direct or indirect roles in cell cycle regulation through cyclin D1, a cyclin expressed early in the cell cycle, and is important for DNA synthesis [49, 50]. Most of these genes are shown to be up-regulated in human prostate cancer, suggesting that inhibition of NF-κB activation might inhibit prostate carcinogenesis. In cell culture studies and in vivo models, apigenin has been shown to inhibit the expression of various cytokines including IL-1β, COX-2, iNOS, VEGF, MMP-9 and cyclin D1 [29, 30]. We have recently demonstrated that apigenin suppresses protein expression of XIAP, c-IAP1, c-IAP2 and survivin in human prostate cancer cells [41]. We found in the present study that apigenin feeding to TRAMP mice suppressed expression of cyclin D1, VEGF, COX-2, Bcl2 and Bcl-xL in the dorso-lateral prostate of TRAMP mice.

Since induction of apoptosis in cancer cells is another strategy for limiting their uncontrolled proliferation, we studied the expression of pro-apoptotic protein Bax and caspase activation as a result of apigenin intake. We found that apigenin intake induces apoptotic death in prostate tumor cells, which was accompanied by higher expression of cleaved caspase 3, a terminal caspase regarded as a marker of apoptotic cell death. In addition, increase in Bax expression observed in the dorsolateral prostates of apigenin-fed TRAMP mice, shifted the Bax/Bcl2 ratio in favor of apoptosis. However, further studies are required to elucidate the mechanism of apoptosis induction by apigenin.

In recent years, cancer researchers have increasingly focused on identifying safe anticancer agents that are found within the spectrum of normal human diets and are therefore acceptable to patients in therapeutic regimens. In this regard, case-control studies have shown a strong inverse association between plant flavone intake and increase risk in the development of breast, colorectal, prostate and epithelial ovarian cancer [51–55]. Numerous studies are available where plant flavones have been proven to be effective as anticancer agents in various experimental models of human cancers and at least a few of them have been tested in clinical trials [28–30, 43, 56–58]. Reports suggest that flavones have ability to inhibit cytochrome (CYP) 450 enzymes, in particular CYP1A1 and CYP1A2 [59]. Recent studies suggest that plant flavones can inhibit CYP3A4, CYP2C9 and p-glycoproteins known to participate in drug metabolism and chemotherapeutic resistance [60, 61]. These studies indicate that inhibition of CYP450 family of enzymes by plant flavones could be used to synergistically enhance the efficacy of chemotherapeutic agents by overcoming acquired resistance in various human cancers. This area of research needs further investigation. Studies have shown that apigenin has limited bio-availability in pure form and is generally present in food bound to sugar moiety as β-glycosides [62]. Apigenin in natural forms is routinely ingested in human diets, and its systemic distribution has been well studied and documented [62, 63]. Upon absorption apigenin is metabolized by methylation or by conjugation with glucornate or sulfate *via* dual recycling involving both enteric and enterohepatic pathways through CYP450 family of enzymes [63, 64]. Knowledge of the pharmacokinetic activities of apigenin in humans is limited. To-date only one clinical trial investigating the possible beneficial effects of apigenin in human subjects has been reported [65]. A study on the bioavailability of apigenin from apiin-rich parsley in healthy human subjects demonstrated that consumption of single oral bolus of 2g of blanched parsley corresponding to 65.8±15.5 μM apigenin per kilogram body weight resulted in 127±81nM/L apigenin plasma concentration after 7.2 h [65]. Our studies on TRAMP mice at 20 and 50 μg/day corresponds to ~50 and ~120 mg/day of flavone intake by an adult human with the plasma apigenin concentration ranging from 0.63 to 1.15 μM/L. These results indicate that readily achievable bioavailable levels of apigenin possess a therapeutic effect. Nonetheless, detailed pharmacokinetic studies on apigenin especially its accumulation in various critical organ sites are needed.

## Conclusions

In summary, the data presented demonstrate that apigenin intake in a mouse model of prostate cancer can suppress NF-κB activation, and its DNA binding reduces the expression of downstream target proteins COX-2, VEGF, cyclin D1, Bcl2 and Bcl-xL resulting in cell cycle arrest and induction of apoptosis in prostate tumors. Our findings suggest that cyclin D1 and Bcl2 expression may be useful as predictive markers of responsiveness of prostate cancer to apigenin therapy. It is notable that the expression of these two NF-κB targets has been identified in other studies as being prognostically important in prostate cancer [66, 67]. Our findings indicate that further evaluation of apigenin as a therapeutic agent in the management of prostate cancer is warranted.
